# A Rare Case of Autoimmune Disorder as a Trigger for Atypical Hemolytic Uremic Syndrome

**DOI:** 10.7759/cureus.53126

**Published:** 2024-01-28

**Authors:** Amit Pasari, Manish Balwani, Prasad Gurjar, Kapil Sejpal, Charulata Bawankule, Priyanka Tolani, Shubham Dubey, Pranjal Kashiv, Amol Bhawane, Sunny Malde, Sushrut Gupta

**Affiliations:** 1 Department of Nephrology, Jawaharlal Nehru Medical College, Datta Meghe Institute of Higher Education and Research, Wardha, IND; 2 Department of Nephrology, Saraswati Kidney Care Center, Nagpur, IND; 3 Department of Internal Medicine, Jawaharlal Nehru Medical College, Datta Meghe Institute of Higher Education and Research, Wardha, IND; 4 Department of Nephrology, All India Institute of Medical Sciences, Nagpur, Nagpur, IND

**Keywords:** anca-associated vasculitis, atypical hus, vasculitis, end-stage kidney disease, renal dysfunction, atypical hemolytic uremic syndrome, antineutrophil cytoplasmic antibody

## Abstract

Autoimmune diseases may act as a trigger for atypical hemolytic uremic syndrome (aHUS). Triggers for aHUS may include autoimmune diseases, infections, metabolic conditions, pregnancy, and transplants. aHUS-mediated injury to various organs, especially kidneys, can be life-threatening. Here, we present the case of a young female who had perinuclear antineutrophil cytoplasmic antibody (p-ANCA)-associated vasculitis and was diagnosed with aHUS. We consider underlying autoimmune p-ANCA-associated vasculitis as a trigger for aHUS in this case.

## Introduction

Atypical hemolytic uremic syndrome (aHUS) is a rare disorder secondary to complement dysregulation. The presence of mutations in complement factors predisposes to development of aHUS. aHUS predominantly affects the kidneys [1]. A trigger-like infection may precipitate the clinical manifestation of aHUS. Apart from infection, surgical stress, pregnancy, and autoimmune disorders may also act as triggers for aHUS. Among the immunological triggers for aHUS, systemic lupus erythematosus, antiphospholipid syndrome, and scleroderma have been reported [2,3]. Here, we present the case of a young female who had perinuclear antineutrophil cytoplasmic antibody (p-ANCA) positivity triggering underlying aHUS.

This case was previously presented at the International Society of Nephrology (ISN) Frontiers Meeting, Bergamo, in 2022.

## Case presentation

**Figure 1:**
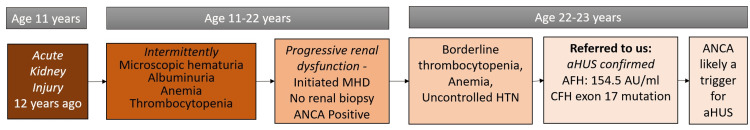
Case flow. ANCA: anti-neutrophilic cytoplasmic antibodies; aHUS: atypical hemolytic uremic syndrome; CFH: *complement factor H* gene; HTN: hypertension; MHD: maintenance hemodialysis

A 23-year-old female, a known case of chronic kidney disease, had an acute kidney injury nearly 12 years ago that was partially recovered. Her medical records (between 13 and 23 years of age) were available for review and revealed the presence of anemia, thrombocytopenia, microscopic hematuria, and proteinuria on multiple occasions. The patient did not consult a nephrologist nor did she undergo a kidney biopsy in the past. She received corticosteroids intermittently from her primary care physician. At 21 years of age, she developed uremic symptoms, for which maintenance hemodialysis was initiated at another local hospital. She had persistently uncontrolled blood pressure despite being on four antihypertensive drugs (nifedipine, metoprolol, telmisartan, and moxonidine). The patient also gave a history of receiving five units of blood transfusions for correction of anemia over the last two years. She presented to our hospital at 23 years of age. At presentation, she had uncontrolled blood pressure (170/90 mmHg) with a history of persistent intra- and inter-dialytic hypertension. Upon a detailed evaluation, she had positive antinuclear antibodies by immunofluorescence and p-ANCA on indirect immunofluorescence. Her anti-myeloperoxidase ANCA antibody (by enzyme-linked immunosorbent assay) was positive (158.02 ng/mL). As the patient’s previous records showed persistent anemia despite receiving adequate erythropoietin, she was also detected to have borderline thrombocytopenia (platelet counts between 90,000 and 110,000/mm^3^) on multiple occasions. Therefore, we had strong clinical suspicion of aHUS. On investigation, anti-factor H antibody levels were found to be substantially elevated at 154.5 AU/mL (normal range: less than 100 AU/mL). Clinical exome sequence analysis showed heterozygous deletion in the *complement factor H* gene (exon 17). Her ADAMTS13 level was normal (0.44 IU/mL). Renal biopsy was not performed given bilateral small kidneys and low yield of histopathology in patients with end-stage renal disease. She is currently following up with us and is on twice-a-week maintenance hemodialysis. She is being managed conservatively without any immunosuppressive regime as aHUS activity is silent. She has plans for a kidney transplant in the future. Figure 1 shows the case flow.

## Discussion

Currently, we have a limited understanding of the renal dysfunction caused by the alterations in complement system pathways. p-ANCA-mediated vasculitis and aHUS co-existence in a single patient is very rare and may be correlated by complement dysregulation and genetic association [4]. aHUS is characterized by acute renal dysfunction, thrombotic microangiopathy, hemolytic anemia, and thrombocytopenia [3,5]. There are multiple triggers for aHUS which include infections, autoimmune disorders, metabolic abnormalities, pregnancy, and organ transplants [5]. Due to the lack of complement regulators in the endothelium of the glomerular capillaries, there is a high risk for complement activation. Podocyte-derived vascular growth factor affects the health of glomerular endothelial cells and injury to podocytes can cause injury to the glomerulus [5]. Control of hypertension is essential in such patients to avoid further injury to the kidney [6]. Severe hypertension with uncontrolled blood pressure has been shown to be associated with rapid progression to end-stage renal dysfunction (ESRD) [7]. aHUS treatment approach requires finding the trigger factor to eliminate it and regulating the complement dysregulation [3,5].

Traditionally, ANCA-associated vasculitis is a challenging diagnosis for physicians, the reason being the low prevalence of ANCA-associated vasculitis. ANCA are produced against the cytoplasmic antigens of neutrophils, especially the white blood cells and the monocytes. The interaction of ANCA with the neutrophils leads to organ-specific outcomes or symptoms. The p-ANCA targets the myeloperoxidase antigen. p-ANCA-associated vascular damage can lead to the development of aHUS [8]. Alternatively, aHUS can trigger the progression of other autoimmune disorders such as p-ANCA-associated vasculitis [9]. Similar to our observation, ANCA-negative renal limited vasculitis and aHUS have been reported in a 40-year-old female patient who developed ESRD [4].

For aHUS, eculizumab is an approved therapy. However, its availability and affordability remain major challenges in a developing country like India. In the absence of eculizumab, plasma exchange may benefit patients with aHUS.

## Conclusions

aHUS is a rare and potentially life-threatening disease. It can lead to ESRD if not treated promptly. Multiple triggers may precipitate aHUS. In this case, we believe ANCA-associated vasculitis acted as a trigger for aHUS. This case highlights the importance of early identification of etiology for renal dysfunction. Timely and appropriate treatment can prevent further deterioration of kidney function.

## References

[1] Sathe KP, Mehta KP (2016). Coexistence of atypical hemolytic uremic syndrome with membranoproliferative glomerulonephritis and antineutrophil cytoplasmic antibodies-associated vasculitis. Saudi J Kidney Dis Transpl.

[2] Rysava R, Peiskerova M, Tesar V (2022). Atypical hemolytic uremic syndrome triggered by mRNA vaccination against SARS-CoV-2: case report. Front Immunol.

[3] Kavanagh D, Goodship TH, Richards A (2013). Atypical hemolytic uremic syndrome. Semin Nephrol.

[4] Mehmood M, Anees M, Ahmad S, Elahi I, Mateen F-, Hussain M, Ashraf S (2021). Coexistence of anti neutrophilic cytoplasmic antibody (ANCA) negative renal limited vasculitis and atypical- hemolytic uremic syndrome (aHUS). Iran J Kidney Dis.

[5] Lee H, Kang E, Kang HG (2020). Consensus regarding diagnosis and management of atypical hemolytic uremic syndrome. Korean J Intern Med.

[6] Zhang B, Xing C, Yu X, Sun B, Zhao X, Qian J (2008). Renal thrombotic microangiopathies induced by severe hypertension. Hypertens Res.

[7] Timmermans SA, Abdul-Hamid MA, Vanderlocht J, Damoiseaux JG, Reutelingsperger CP, van Paassen P (2017). Patients with hypertension-associated thrombotic microangiopathy may present with complement abnormalities. Kidney Int.

[8] Hu T, Dattani ND, Pagnoux C, Jain R (2016). Perinuclear antineutrophil cytoplasmic antibody-associated vasculitis in an elderly woman. Can Fam Physician.

[9] Cavero T, Rabasco C, López A (2017). Eculizumab in secondary atypical haemolytic uraemic syndrome. Nephrol Dial Transplant.

